# Supplementary material for: Longitudinal course of inflammatory-cognitive subgroups across first treatment severe mental illness and healthy controls
Source: Psychol Med. 2024 Oct 2;54(12):3519–29. doi: 10.1017/S003329172400206X (PMC11496234; doi:10.1017/S003329172400206X)
Supplement: Sæther et al. supplementary material 2 — Sæther et al. supplementary material [file S003329172400206Xsup002.docx]

**Supplementary Methods**

1. **R-packages used for visualization and main statistical analyses**

- For visualization the R-package “ggplot2” was applied (Wickham, 2016).
- For permutation-based t-tests (sample & clinical characteristics) the R-package “rcompanion” (Mangiafico, 2022) was used.
- Clustering was performed using R-packages “cluster” (Maechler et al., 2022), “dendextend” (Galili, 2015) and “factoextra” (Kassambara and Mundt, 2020).
- Clustering stability was assessed using the R-package “fpc” (flexible procedures for clustering) (Hennig, 2020).
- Linear mixed models were run using “lme4" (Bates et al., 2015), “lmerTest” (Kuznetsova et al., 2017) and model comparisons with “emmeans” (Lenth et al., 2018).
- Tables were automatically generated using “gtsummary” (Sjoberg et al., 2021).

**References**

Bates, D., Mächler, M., Bolker, B., Walker, S., 2015. Fitting Linear Mixed-Effects Models Using lme4. Journal of Statistical Software 67, 1–48. https://doi.org/10.18637/jss.v067.i01

Galili, T., 2015. dendextend: an R package for visualizing, adjusting and comparing trees of hierarchical clustering. Bioinformatics 31, 3718–3720. https://doi.org/10.1093/bioinformatics/btv428

Hennig, C., 2020. fpc: Flexible Procedures for Clustering. R package version 2.2-9.

Kassambara, A., Mundt, F., 2020. factoextra: Extract and Visualize the Results of Multivariate Data Analyses. R package version 1.0.7.

Kuznetsova, A., Brockhoff, P.B., Christensen, R.H.B., 2017. lmerTest Package: Tests in Linear Mixed Effects Models. Journal of Statistical Software 82, 1–26. https://doi.org/10.18637/jss.v082.i13

Lenth, R., Singmann, H., Love, J., Buerkner, P., Herve, M., 2018. Package “Emmeans”. R Package Version 4.0-3.

Maechler, M., Rousseeuw, P., Struyf, A., Hubert, M., Hornik, K., 2022. cluster: Cluster Analysis Basics and Extensions. R package version 2.1.3.

Mangiafico, S., 2022. rcompanion: Functions to Support Extension Education Program Evaluation. R package version 2.4.15.

Sjoberg, D.D., Whiting, K., Curry, M., Lavery, J.A., Larmarange, J., 2021. Reproducible Summary Tables with the gtsummary Package. The R Journal 13, 570–580.

Wickham, H., 2016. ggplot2: Elegant Graphics for Data Analysis. Springer.
